# Diagnostic accuracy of Truenat MTB Plus, Truenat MTB Ultima and Xpert MTB/RIF Ultra for the diagnosis of pulmonary TB in an HIV-endemic setting

**DOI:** 10.21203/rs.3.rs-5055991/v1

**Published:** 2024-09-10

**Authors:** Shima M Abdulgader, Arthur M Chiwaya, Byron W P Reeve, Zaida Palmer, Hridesh Mishra, Desiree L Mbu, Nondumiso Lushozi, Zola Nkwanyana, Morten Ruhwald, Adam Penn-Nicholson, Robin Warren, Grant Theron

**Affiliations:** DSI-NRF Centre of Excellence for Biomedical Tuberculosis Research; South African Medical Research Council Centre for Tuberculosis Research; Division of Molecular Biology and Human Genetics, Faculty of Medicine and Health Sciences, Stellenbosch University, Cape Town, South Africa; DSI-NRF Centre of Excellence for Biomedical Tuberculosis Research; South African Medical Research Council Centre for Tuberculosis Research; Division of Molecular Biology and Human Genetics, Faculty of Medicine and Health Sciences, Stellenbosch University, Cape Town, South Africa; DSI-NRF Centre of Excellence for Biomedical Tuberculosis Research; South African Medical Research Council Centre for Tuberculosis Research; Division of Molecular Biology and Human Genetics, Faculty of Medicine and Health Sciences, Stellenbosch University, Cape Town, South Africa; DSI-NRF Centre of Excellence for Biomedical Tuberculosis Research; South African Medical Research Council Centre for Tuberculosis Research; Division of Molecular Biology and Human Genetics, Faculty of Medicine and Health Sciences, Stellenbosch University, Cape Town, South Africa; DSI-NRF Centre of Excellence for Biomedical Tuberculosis Research; South African Medical Research Council Centre for Tuberculosis Research; Division of Molecular Biology and Human Genetics, Faculty of Medicine and Health Sciences, Stellenbosch University, Cape Town, South Africa; DSI-NRF Centre of Excellence for Biomedical Tuberculosis Research; South African Medical Research Council Centre for Tuberculosis Research; Division of Molecular Biology and Human Genetics, Faculty of Medicine and Health Sciences, Stellenbosch University, Cape Town, South Africa; DSI-NRF Centre of Excellence for Biomedical Tuberculosis Research; South African Medical Research Council Centre for Tuberculosis Research; Division of Molecular Biology and Human Genetics, Faculty of Medicine and Health Sciences, Stellenbosch University, Cape Town, South Africa; DSI-NRF Centre of Excellence for Biomedical Tuberculosis Research; South African Medical Research Council Centre for Tuberculosis Research; Division of Molecular Biology and Human Genetics, Faculty of Medicine and Health Sciences, Stellenbosch University, Cape Town, South Africa; Foundation for Innovative New Diagnostics: FIND; Foundation for Innovative New Diagnostics: FIND; DSI-NRF Centre of Excellence for Biomedical Tuberculosis Research; South African Medical Research Council Centre for Tuberculosis Research; Division of Molecular Biology and Human Genetics, Faculty of Medicine and Health Sciences, Stellenbosch University, Cape Town, South Africa; DSI-NRF Centre of Excellence for Biomedical Tuberculosis Research; South African Medical Research Council Centre for Tuberculosis Research; Division of Molecular Biology and Human Genetics, Faculty of Medicine and Health Sciences, Stellenbosch University, Cape Town, South Africa

**Keywords:** Tuberculosis, Diagnosis, HIV, Ultra, Truenat

## Abstract

**Background:**

Truenat MTB Plus (MTB Plus) and MTB Ultima (Ultima) are World Health Organization-endorsed low-complexity tuberculosis (TB) tests, however, performance data are scarce.

**Methods:**

Adults (≥18 years; n=498) self-presenting with symptoms to primary care clinics in Cape Town, South Africa (19/02/2016–22/02/2023) provided sputa. We evaluated the accuracy of MTB Plus and Ultima, with Xpert MTB/RIF Ultra (Ultra) as a comparator, vs. a single culture (TB reference standard) or MTBDR*plus* on an isolate (rifampicin susceptibility reference standard).

**Results:**

The proportion of MTB Plus and Ultima unsuccessful results was 20% (95% confidence interval 17, 23) and 14 (11, 16), respectively, with ≥half resolving upon retesting the same eluate. In a three-way analysis, MTB Plus, Ultima and Ultra had sensitivities of 84% (78, 88), 90% (85, 93), and 92% (87, 95), and specificities of 95% (92, 97), 85% (80, 88) and 95% (92, 97) for TB. The proportion of unsuccessful results for MTB-RIF Dx done the same day as DNA extraction was 9% (3, 16; MTB Plus-positives) and 18% (10, 26; Ultima-positives) [if after day-of-extraction, these were 27% (18, 35) and 44% (35, 51)]. Same-day rifampicin susceptibility testing was often unsuccessful in samples with “very low” load [73% (58, 89) MTB Plus, 75% (65, 86) Ultima] but had 100% (40, 100) sensitivity and 99% (96, 100) specificity (for both MTB Plus- or Ultima-positive DNA). Lot variation in unsuccessful and false-positive results was observed.

**Conclusion:**

Ultima showed comparable sensitivity to Ultra but specificity, lot variation, and, like MTB-RIF Dx, unsuccessful result rates were suboptimal.

**Funding:**

European & Developing Countries Clinical Trials Partnership, and South African Medical Research Council.

## Introduction

Rapid diagnostics and treatment can reduce global tuberculosis (TB) incidence (1). In 2022, ~30% of the 10.6 million new TB cases were undiagnosed, driving transmission (1). Better diagnostics, including cheaper tests, are key to addressing this care cascade gap.

Xpert MTB/RIF Ultra (Ultra; Cepheid, Sunnyvale, USA), a WHO-recommended confirmatory nucleic acid amplification test (NAAT) for *Mycobacterium tuberculosis* complex (MTBC), targets *rpoB, IS6110* and *IS1081*, with 91% sensitivity in people with presumptive TB (78% in smear-negative people) (2). Ultra has, due to free DNA associated with non-intact cells, suboptimal specificity in people with previous TB (3, 4), which has resulted in countries changing their diagnostic algorithms (5). Alternative tests may be less affected by old DNA or DNA associated with non-intact cells. Furthermore, Ultra and its required equipment is, even with concessional pricing, financially out-of-reach for many TB programmes (6, 7). This, compounded with stock outs and overreliance on a single manufacturer, motivated the development of fast-follower technologies.

Truenat MTB Plus (MTB Plus; Goa, India) is a low-complexity NAAT that utilises chip-based real-time PCR for TB detection by amplifying *nrdZ* and *IS6110* (8).It involves a two-step process where DNA extraction is first done using the Trueprep instrument, DNA is manually transferred into a PCR tube then placed into a separate Truelab instrument. Truenat devices are portable and battery-operated (9). MTB Plus was WHO-endorsed in 2020 for sputum, however, it was noted more data were needed especially from people living with HIV (PLHIV), for whom data was extrapolated from other sources (10). A recent study including hospitalised PLHIV reported 85% MTB Plus sensitivity (55% in smear-negatives) (11).

Molbio recently developed the Truenat MTB Ultima (Ultima), which additionally includes *IS1081* (12). Ultima is not WHO endorsed but is designed to provide superior sensitivity to existing products (13). There are published performance data of Ultima only on tongue swabs, where it had 71% sensitivity and 97% specificity (Ultra had 76% sensitivity and 100% specificity) (14).

Truenat MTB-RIF Dx (MTB-RIF Dx) is a reflex test to amplify *rpoB* in the same sputum DNA eluate detected as TB positive by MTB Plus or Ultima. MTB-RIF Dx is WHO-endorsed with 84% sensitivity and 97% specificity when reflexed from MTB Plus-positive DNA (10). DNA detected as positive by Ultima, which may not have been detected by MTB Plus, may result in differences in MTB-RIF Dx performance.

Our objective was to therefore assess, in our high burden country of South Africa where HIV is frequent and many people have previous TB, the accuracy of Ultima on sputum for the diagnosis of pulmonary TB in people presenting to care, with MTB Plus and Ultra as comparators. We also assessed MTB-RIF Dx in people diagnosed with TB. We hypothesised that Ultima would have similar performance to Ultra.

## Methods

### Ethics

The study was conducted in accordance with the Declaration of Helsinki and was approved by Stellenbosch University Faculty of Health Sciences Research Ethics Committee (N14/10/136) and the City of Cape Town (6470). People provided a written informed consent.

### Study design, participant flow and specimen collection

**Figure 1:**
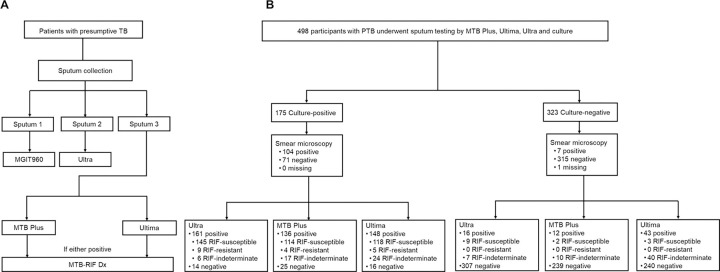
(A) Specimen and (B) participant selection and test results for TB detection. Participants with successful Ultra and Truenat results were included in the diagnostic accuracy analyses. Abbreviations: MGIT960, Mycobacteria Growth Indicator Tube; MTB, *Mycobacteria tuberculosis*; RIF, Rifampicin; Ultra, Xpert MTB/RIF Ultra.

Adults (≥18 years) self-presenting with presumptive TB (15), not on treatment currently or within the last two months were consecutively recruited between 19 February 2016 and 22 February 2023 at primary care clinics in Cape Town, South Africa. Demographic, clinical, and microbiological data were captured on REDCap (16). Previous TB status was obtained via questionnaire. Three sputa specimens were provided over two consecutive workdays (Figure 1). Participants unable to expectorate were induced as described (17). To enable head-to-head comparisons with MTB Plus and Ultima tests, only sputa from people with successful Ultra and culture results (positive or negative) were selected.

### Sputum microbiology

#### Smear microscopy and culture

Each participant provided at least three sputum samples. The most viscous (visually determined) sputum was used for double Ziehl-Neelsen smear microscopy and a MGIT960 liquid culture (BD, Franklin Lakes, USA). The remaining two samples were arbitrarily selected for Ultra (done fresh) or Truenat tests (done after biobanking involving storage at −80°C) (Figure 1). GenoType MTBDR*plus* (v2.0; Bruker-Hain Life Sciences, Nehren, Germany) was done on culture-positive growth for MTBC and rifampicin resistance detection.

#### Ultra

Xpert MTB/RIF Ultra (v2; Cepheid, Sunnyvale, USA) was done on raw sputum per the manufacturer’s protocol (18). In brief, 1.4 ml sample reagent (SR) buffer was added to 700 μl sputum, the mixture incubated (room temperature, 10 min) and 2 mL transferred into the cartridge.

#### Truenat MTB Plus, Ultima, and MTB-RIF Dx

Truenat tests (Molbio, Goa, India) were done between 01/11/ 2022–05/06/2023 per the manufacturer’s instructions (12, 19, 20). In brief, 0.5 mL of thawed sputum was added to 2.5 mL specimen reagent (lysis buffer). The mixture (3 ml) was loaded on the Trueprep (v2) instrument for DNA extraction, resulting in ≥100 μl eluate. Immediately upon extraction, 6 μl eluate was used for each MTB Plus and Ultima. A test was repeated on the eluate if that test’s first result was unsuccessful (invalid or error). MTB-RIF Dx was performed, either after biobanking and thawing (n=127) or same day [n=80; after an update to the instructions for use were issued (21)], on MTB Plus- or Ultima-positive DNA using the same eluate. No repeat testing was done for MTB-RIF Dx unsuccessful eluates. Two percent (9/504) of people were erroneously double tested using MTB Plus (n=4) and Ultima (n=5) and, for each test, one result from each person was randomly selected. Clinical information and reference standard results were unavailable to test operators. The MTB Plus, Ultima, and MTB-RIF Dx test results were categorized as successful (MTBC detected or not detected, and RIF resistance detected or not detected, respectively) or unsuccessful (invalid, error or indeterminate, on Truelab).

### Definitions

#### Microbiological Reference Standard (MRS)

A definite TB case was defined as MGIT960 culture-positive sputum (with MTBC confirmation). A non-TB participant was defined as culture-negative. Participants were unclassifiable if sputum culture was either contaminated or positive without MTBC speciation. The extended microbiological reference standard (eMRS) includes Ultra. More information and other definitions are in the supplement.

### Statistical analysis

STARD guidelines were followed (supplementary pg. 17) (22). Sensitivity, specificity, and predictive values of MTB Plus, Ultima, and Ultra were calculated using 2×2 tables versus the MRS or eMRS. All participants had each test attempted (MTB Plus, Ultima, Ultra) and all comparisons involving these tests are therefore head-to-head. We present index test results without retesting unless specified otherwise. Diagnostic yield metrics (DYT, diagnostic yield in those tested; DYD, diagnostic yield in those diagnosed) were calculated as described (23). We analysed data using the χ^2^ test (including McNemar’s test) and the two-sample proportion test (24). We used Stata (v18; StataCorp LLC, USA) and GraphPad Prism (v8; GraphPad Software, USA).

## Results

### Demographic and clinical characteristics

Of 498 adults, 35% (175) were TB cases according to MRS, 43% (213) were female, 54% (269) PLHIV, and 65% (323) current or past smokers (Table 1). People with TB were younger, had lower CD4 counts if HIV-positive, greater morbidity (TBscoreII) and lower haemoglobin than non-TB participants.

### Unsuccessful TB results

The proportion of unsuccessful results for MTB Plus and Ultima were 17% (86/501) (95% CI 14, 20) and 10% (52/501) (8,13; p=0.0018), respectively (**Supplementary table 1**; majority invalids). No temporal associations were detected for unsuccessful Truenat results (**Supplementary figure 3**), however, we noted unsuccessful results inter-lot variation occurred for Ultima (and, for MTB Plus, false positivity) (**Supplementary table 2**).

### Diagnostic accuracy for TB

MTB Plus sensitivity was 84% (78, 88) and specificity 95% (92, 97), with sensitivity decreasing to 68% (55, 77) in smear-negatives. Ultima sensitivity was 90% (85, 93) and specificity 85% (80, 88), with sensitivity decreasing to 80% (68, 87) in smear-negatives. Sensitivity did not differ by HIV status for MTB Plus and Ultima. MTB Plus showed a decreased specificity in HIV-negative people vs. PLHIV [92% (85, 95) vs. 98% (94, 99); p=0.0237]. Ultra sensitivity was 92% (95% CI 87, 95) and specificity 95% (92, 97), with sensitivity decreasing to 80% (69, 87) in smear-negative participants (Table 2). Specificity for MTB Plus, Ultima and Ultra were unaffected by previous TB (Table 3). Estimates did not differ significantly when eMRS was used, especially Ultima’s specificity (**Supplementary table 3**).

### Repeat testing of unsuccessful results

Of the MTB Plus unsuccessful results, 60% (52/86) resolved after repeat testing (12 became positive, 40 negatives; remainder remained unsuccessful). Of the Ultima unsuccessful results, 54% (28/52) resolved upon repeat testing (6 became positive, 22 negatives; remainder remained unsuccessful) (**Supplementary table 1**). Repeat testing increased the yield of positive results by 2% (30–32%) for MTB Plus (148 to 160) and 2% (38–40%) for Ultima (191 to 200) (**Supplementary table 4**)

### Rifampicin resistance

#### Unsuccessful results:

208 MTB-RIF Dx tests were done (138 positive on both MTB Plus and Ultima, 10 MTB Plus-positive Ultima-negative, 53 Ultima-positive MTB Plus-negative (**Supplementary table 5**). In MTB Plus-positive participants, the proportion unsuccessful MTB-RIF Dx results was 19% (28/148) and, for Ultima-positive participants 34% (65/191). Ultra’s overall unsuccessful result rate for rifampicin susceptibility was 12% (44/360). When results were stratified by whether MTB-RIF Dx was done on biobanked eluates or tested same day, the proportion of unsuccessful results decreased from 27% (22/82) to 9% (6/66) for MTB Plus-positive eluates and from 44% (51/115) to 18% (14/76) for Ultima-positive eluates. Most unsuccessful results were in the “very low” semi-quantitation categories for MTB Plus- and Ultima [73% (22/30) and 75% (52/69), respectively; **Supplementary table 6**].

#### Sensitivity and specificity:

MTB-RIF Dx sensitivity and specificity was 100% (4/4) and 99% (125/126) whether done on MTB Plus- or Ultima-positive eluates. Ultra had a sensitivity and specificity of 100% (4/4) and 97% (145/150; **Supplementary table 7**).

### Cases potentially missed in a hypothetical population

#### TB:

Applying the overall unsuccessful and false negative rates seen in this study, in 1000 smear-negative cases, MTB Plus and Ultima would each miss 200 cases (MTB Plus: 170 unsuccessful, 30 false-negatives; Ultima: 100 unsuccessful, 100 false-negatives). With repeat testing of those initially unsuccessfully, missed cases would reduce to 100 (70 still unsuccessful, 30 false-negatives) for MTB Plus and 151 (50 unsuccessful, 101 false-negatives) for Ultima.

#### Rifampicin resistance:

Assuming the 1000 smear-negative cases had rifampicin resistance and immediate rifampicin testing done using MTB-RIF Dx, of the 800 correctly diagnosed as TB-positive by MTB-Plus (without repeat of unsuccessful results), 72 would be missed (all MTB-RIF Dx unsuccessful, assuming MTB-RIF Dx had 100% sensitivity for rifampicin resistance). For MTB-RIF Dx reflexed from Ultima-positive, 144 would be missed (all MTB-RIF Dx unsuccessful, assuming MTB-RIF Dx had 100% sensitivity for rifampicin resistance). However, patients with those phenotypic characteristics might experience different unsuccessful and false negative rates.

## Discussion

We evaluated the accuracy of Ultima in comparison to MTB Plus and Ultra on sputum from people with symptoms of TB in a high HIV and TB burden setting. Our key findings are: 1) the proportion of unsuccessful results are significant (~10% with Ultima) and result in missed TB diagnoses, however, retesting halves the number of participants who do not receive a result, 2) Ultima sensitivity was similar to that of MTB Plus and Ultra, 3) Ultima specificity was low (~85%), resulting in approximately 1 in 5 positive results being false-positive (not associated with previous TB), 4) lot variation in MTB Plus and Ultima performance was observed, and 5) MTB-RIF Dx must be done immediately on eluted DNA, has approximately double (19%) the unsuccessful rate on Ultima-positive rather than MTB Plus-positive DNA (even when done fresh) and, due to a low probability of success, should not be done on samples on samples with an MTB Plus or Ultima semi-quantitation classification of “very low”. Together, these data have implications for Truenat adoption.

There are, to our knowledge, no published data of Ultima’s accuracy on sputum. In addition to Ultima, our study increases the evidence base for MTB Plus, in whom data from PLHIV and people with a history of TB was scarce (8, 11). Although we pre-selected specimens from participants with successful Ultra and culture results, a noteworthy proportion of unsuccessful results prior to repeat testing for MTB Plus (17%) and Ultima (10%) were observed consistently over the testing period without any temporal association. This parallels other studies (8, 11, 25), that reported invalid MTB Plus results from 9–18%. Importantly, we show that more TB cases are missed due to the test being unsuccessful rather than false-negative, highlighting the importance of quantifying unsuccessful results in test evaluations, something recently highlighted in the recently updated WHO TPP, where an acceptable unsuccessful result rate was defined as 3–5% (26).

Molbio recommends retesting using the same eluate when the initial TB result is unsuccessful (12, 19). This is supported by our data as, upon retesting, 60% of MTB Plus eluates initially unsuccessful became successful (54% for Ultima). Repeating would increase people diagnosed who might not otherwise return to give another sputum. Reasons why our retesting of the same eluate had success may be because, at initial testing, the DNA eluate was not sufficiently suspended with the lyophilised pellet containing PCR reagents. Molbio recommends allowing this mixture to stand for 30–60 seconds to achieve a clear solution before proceeding (12, 19), however, as the only factor that differed upon retesting was time, the manufacturer should consider extending the duration of standing. Before adopting retesting, laboratories would need to factor in cost and workload.

MTB Plus and Ultima had 84% and 90% sensitivity compared to 92% for Ultra. Our MTB Plus sensitivity estimate is like others in high HIV-prevalence settings. Among all participants, sensitivity was similar between MTB Plus and Ultima, but Ultima had higher sensitivity than MTB Plus in PLHIV. Ultra sensitivity was higher than MTB Plus for all participants, consistent with previous findings from Peru (8), but there was no difference among PLHIV. These data address the shortage of MTB Plus and Ultima data in PLHIV.

Ultima had lower specificity compared to Ultra, which has similar amplification targets (*IS6110, IS1081*). This is despite both Ultima and MTB Plus (which did not show low specificity in the same people despite also has a step where the tube is open) being done in parallel at the same time and in the same quality-assured laboratory. Importantly, this finding persisted when Ultima was evaluated against an eMRS that included Ultra. Furthermore, unlike what we described before for Xpert and Ultra (4, 27, 28), diminished specificity was not associated with previous TB. This specificity finding, which translates into low PPV for Ultima even in our high burden setting (more than 3/10 positives false-positive per MRS, 2/10 per eMRS), necessitates further investigation, especially if Ultima is to be applied in settings where pre-test probability of disease is lower. In the only other comparison of Ultra and Ultima (on tongue swabs), Ultima specificity was lower than that of Ultra.

We noted clinically important performance variation for MTB Plus and Ultima associated with lot number, both in terms of unsuccessful results and false positivity. Similar challenges have been reported for the SILVAMP TB-LAM test (FujiLAM; Fujifilm, Tokyo, Japan), which led to the test’s postponement (29, 30). Critically, stratification of performance data by lot is not in TB study guidance (31) nor part of the STARD criteria (22). Our data suggest this is important to incorporate, including in evidence review processes for policy making. Lastly, the variation in Ultima lot performance may be due to the product not yet being commercially available. Tightening of manufacturer quality control processes may be needed.

Our study’s primary purpose was not to assess MTB-RIF Dx’s sensitivity for rifampicin susceptibility, which requires further evaluation in people with presumed drug-resistant TB, however, we showed that, when MTB-RIF Dx is applied to Ultima-positive rather than MTB Plus-positive eluates, unsuccessful results are more likely (almost all Ultima-positive “very lows” were MTB-RIF Dx unsuccessful). This is likely because such people were positive exclusively based on the amplification of the multicopy gene target (*IS1081*) that MTB Plus (and MTB-RIF Dx) does not include. Lastly, it remains possible that, as for TB detection, MTB-RIF unsuccessful results may partly resolve upon retesting and, although we did not evaluate this, such a strategy would need to factor in elevated risk of unsuccessful results associated with non-same day testing.

This study addresses a critical research gap by evaluating new and existing Truenat tests for TB detection in a cohort with many PLHIV (the largest to date). Limitations include the use of biobanked samples for Truenat testing. Truenat samples with unsuccessful results were repeated from the same DNA eluate, however, although Molbio recommends repeating the test on a fresh sample, we show repeating from the same DNA eluate is useful (our approach is likely more feasible in situations where specimen re-collection is unfeasible). Although testing was performed in well-resourced research setting with machines calibrated according to the manufacturers’ recommendations and, in the case of the Cepheid and Molbio tests, the tests done years apart, we experienced high rates of unsuccessful results for MTB Plus and Ultima even though sputa from people with an unsuccessful Ultra result were excluded. Further monitoring and research into the extent of these unsuccessful results is required, including in different settings.

In summary, Truenat MTB Plus and Ultima are alternative TB sputum test that met WHO's minimum sensitivity threshold for sputum-based tests for culture-positive TB. Ultima has improved sensitivity compared to MTB Plus in PLHIV. However, Ultima’s suboptimal specificity, lot variation, and the relatively high proportion of unsuccessful results (also for same-day MTB RIF Dx testing) require careful further investigation.

## Figures and Tables

**Figure 2:**
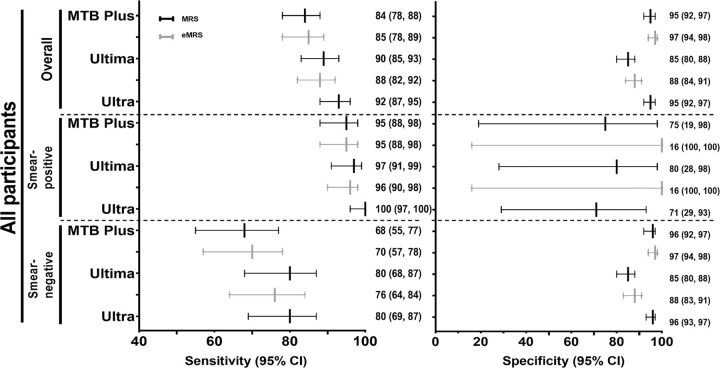
Diagnostic accuracy MTB Plus and Ultima, compared to Ultra, for TB detection versus the MRS and eMRS. The sensitivity of MTB Plus and Ultima did not differ when the MRS and eMRS were used, and Ultima specificity remained suboptimal. Abbreviations: TB, tuberculosis; MRS, microbiological reference standard; eMRS, extended microbiological reference standard.

**Figure 3:**
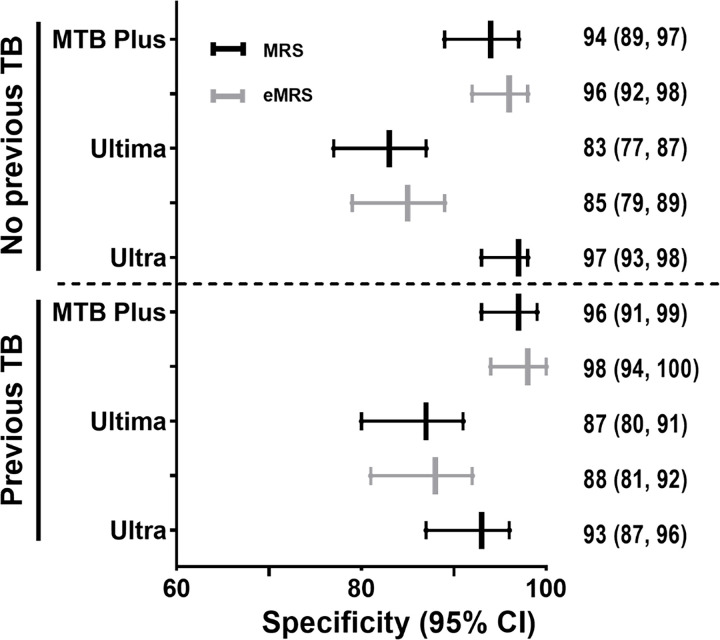
Specificity of MTB Plus and Ultima, compared to Ultra, for TB detection stratified by MRS and eMRS status and previous TB status. Ultima had reduced specificity compared to Ultra and MTB Plus in participants with (n=166) and without previous TB. Abbreviations: TB, tuberculosis; MRS, microbiological reference standard; eMRS, extended microbiological reference standard.

**Table 1.** Demographic and clinical characteristics stratified by TB and smear statuses. Data are median (IQR) or n/N (%).

	Overall n=498	Culture-positive			Culture-negative 323/498 (65)
		All 175/498 (35)	Smear-positive 104/175 (59)	Smear-negative 71/175 (41)	
**Demographic characteristics**					
Age (years)	35 (28–44)	33 (26–41)	33 (27–39)	35 (25–44)	37 (29–46)
				p=0.4441^†^	**p=0.0011** ^‡^
Female	213/498 (43)	70/175 (40)	38/104 (37)	32/71 (45)	143/323 (44)
				p=0.3582^†^	p=0.8167^‡^
Tobacco smoker (past or current)	323/498 (65)	129/175 (74)	81/104 (79)	48/71 (68)	194/323 (60)
				p=0.0775^†^	**p=0.0063** ^‡^
**Clinical characteristics**					
HIV status					
Positive	269/498 (54)	85/175 (49)	50/104 (48)	35/71 (49)	184/323 (57)
				p=0.3207^†^	**p=0.0397** ^‡^
CD4 count (cells/μl)	2 (0–333)	0 (0–241)	0 (0–256)	0 (0–201)	19 (0–377)
				p=0.8848^†^	**p=0.0326** ^‡^
TBscoreII	2 (2–3)	3 (2–4)	3 (2–4)	3 (2–4)	2 (2–3)
				p=0.1116^‡^	**p<0.0001** ^‡^
Haemoglobin (g/dl)	13.2 (11.5–14.7)	12.2 (10.4–13.5)	12.1 (10.4–13.4)	12.4 (10.4–13.8)	13.7 (12.4–15)
				p=0.5858^†^	**p<0.0001** ^‡^
Previous TB	210/498 (42)	71/175 (41)	44/104 (42)	27/71 (38)	139/323 (43)
				p=0.8491^†^	p=0.7879^‡^

‡ P-values comparing all culture-positives to -negatives.

† P-values within culture-positives comparing smear-positives to -negative.

Missing data: smear (n=1), TBscorell (n=6).

Abbreviations: IQR, interquartile range; TBscoreII, TB symptom score II. Bold font represents p values ≤0.05.

**Table 2.** Diagnostic accuracy of smear microscopy, MTB Plus, Ultima and Ultra versus the MRS, with stratification by HIV status. MTB Plus had lower sensitivity than Ultra whereas Ultima was similar. We did not detect sensitivity differences by HIV status. Data are % (95% CI) n/N.

	All participants, n=498				HIV-negative, 229/498 (46%)				HIV-posttive, 269/498 (54%)			
	Sensitivity	Specificity	PPV	NPV	Sensitivity	Specificity	PPV	NPV	Sensitivity	Specificity	PPV	NPV
**Smear microscopy**	59 (52, 65) 104/175	98 (96, 99) 315/322	94 (87, 97) 104/111	82 (77, 84) 71/386	60 (49, 68) 54/90	96 (92, 98) 134/139	92 (81, 96) 54/59)	79 (72, 83) 36/170	59 (48, 67) 50/85	99 (96, 100) 181/183	96 (87, 99) 50/52	84 (78, 87) 35/216
**MTB Plus**	84 (78, 88) 136/161	95 (92, 97) 239/251	92 (86, 95) 136/148	91 (86, 93) 239/264	86 (76, 91) 72/84	92 (85, 95) 100/109	89 (80, 94) 72/81	89 (82, 93) 100/112	83 (73, 89) 64/77	98 (94, 99) 139/142	96 (87, 99) 64/67	91 (86, 95) 139/152
									p=0.1853*	**p=0.0237** *	p=0.7716*	**p=0.0343** *
**Smear-positive**	95 (88, 98) 93/98	75 (19, 98) 3/4	99 (94, 100) 93/94	38 (9, 68) 3/8	96 (87, 99) 49/51	75 (19, 98) 3/4	98 (89, 100) 49/50	60 (15, 90) 3/5	94 (82, 98) 44/47	Non-calculable	100 (92, 100) 44/44	0 (0, 58) 0/3
**Smear-negative**	68 (55, 77) 43/63	96 (92, 97) 235/246	80 (66, 87) 43/54	92 (88, 94) 235/255	70 (51, 81) 23/33	92 (86, 96) 97/105	74 (55, 85) 23/31	91 (83, 94) 97/107	67 (47, 79) 20/30	98 (94, 99) 138/141	87 (66, 96) 20/23	93 (88, 96) 138/148
									p=0.7964*	**p=0.0393** *	p=0.2495*	p=0.4479*
**Ultima**	90 (85, 93) 148/164	85 (80, 88) 240/283	77 (71, 82) 148/191	94 (90, 96) 240/256	87 (79, 92) 76/87	83 (75, 87) 100/121	78 (69, 84) 76/97	90 (83, 94) 100/111	94 (85, 97) 72/77	86 (80, 90) 140/162	77 (67, 83) 72/94	97 (92, 98) 140/145
									p=0.1853*	p=0.3814*	p=0.7716*	**p=0.0343** *
	p=0.1171^†^	**p<0.0001** ^†^	**p=0.0004** ^†^	p=0.1732^†^	p=0.7531^†^	**p=0.0408** ^†^	p=0.0614^†^	p=0.8435^†^	**p=0.0448** ^†^	**p=0.0003** ^†^	**p=0.0011** ^†^	p=0.0654^†^
**Smear-positive**	97 (91, 99) 97/100	80 (28, 98) 4/5	99 (94, 100) 97/98	57 (18, 85) 4/7	98 (90, 100) 52/53	75 (19, 98) 3/4	98 (90, 100) 52/53	75 (19, 98) 3/4	96 (85, 99) 45/47	100 (3, 100) 1/1	100 (92, 100) 45/45	33 (1, 83) 1/3
**Smear-negative**	80 (68, 87) 51/64	85 (80, 88) 235/277	55 (44, 63) 51/93	95 (91, 97) 235/248	71 (53, 82) 24/34	83 (75, 88) 97/117	55 (39, 66) 24/44	91 (83, 94) 97/107	90 (73, 97) 27/30	86 (80, 90) 138/160	55 (40, 66) 27/49	98 (94, 99) 138/141
									p=0.0541*	p=0.4434*	p=0.9571*	**p=0.0115** *
	p=0.1418^†^	**p<0.0001** ^†^	**p=0.0025** ^†^	p=0.2388^†^	p=0.9365^†^	**p=0.0337** ^†^	p=0.0832^†^	p>0.9999^†^	**p=0.0283** ^†^	**p=0.0003** ^†^	**p=0.0081** ^†^	p=0.0577^†^
Ultra**	92 (87, 95) 161/175	95 (92, 97) 307/323	91 (86, 94) 161/177	96 (93, 97) 307/321	92 (85, 96) 83/90	93 (87, 96) 129/139	89 (81, 94) 83/93	95 (90, 97) 129/136	92 (84, 96) 78/85	97 (93, 98) 178/184	93 (85, 96) 78/84	96 (92, 98) 178/185
									p=0.9112*	p=0.1067*	p=0.4030*	p=0.5545*
	p=0.0314^†^	p=0.9241^†^	p=0.7657^†^	**p=0.0137** ^†^	p=0.1690^†^	p=0.7548^†^	p=0.9397^†^	p=0.1009^†^	p=0.0947^†^	p=0.5304^†^	p=0.4919^†^	p=0.0652^†^
	p=0.5694^¶^	**p<0.0001** ^¶^	**p=0.0004** ^¶^	p=0.3100^¶^	p=0.2843^¶^	**p=0.0117** ^¶^	**p=0.0422** ^¶^	p=0.1520^¶^	p=0.6725^¶^	**p=0.0004** ^¶^	**p=0.0029** ^¶^	p=0.8716^¶^
**Smear-positive**	100 (97, 100) 104/104	71 (29, 93) 5/7	98 (93, 100) 104/106	100 (48, 100) 5/5	100 (93, 100) 54/54	60 (15, 90) 3/5	96 (88, 99) 54/56	100 (29, 100) 3/3	100 (93, 100) 50/50	100 (16, 100) 2/2	100 (93, 100) 50/50	100 (16, 100) 2/2
**Smear-negative**	80 (69, 87) 57/71	96 (93, 97) 301/315	80 (69, 87) 57/71	96 (93, 97) 301/315	81 (64, 90) 29/36	94 (89, 97) 126/134	78 (62, 88) 29/37	95 (89, 97) 126/133	80 (63, 89) 28/35	97 (93, 98) 175/181	82 (65, 91) 28/34	96 (92, 98) 175/182
									p=0.9531*	p=0.2585*	p=0.6741*	p=0.5467*
	p=0.1103^†^	p=0.9877^†^	p=0.9281^†^	p=0.0885^†^	p=0.2957^†^	p=0.6127^†^	p=0.6853^†^	p=0.2204^†^	p=0.2227^†^	p=0.5214^†^	p=0.6401^†^	p=0.2342^†^
	p=0.9313^¶^	**p<0.0001** ^¶^	**p=0.0007** ^¶^	p=0.6602^¶^	p=0.3311^¶^	**p=0.0052** ^¶^	**p=0.0247** ^¶^	p=0.2204^¶^	p=0.2653^¶^	**p=0.0005** ^¶^	**p=0.0098** ^¶^	p=0.3765^¶^

Within row p-values:

* HIV-positive vs. HIV-negative.

Within column p-values for people of the same smear status:

† vs. MTB Plus

¶ vs. Ultima

Abbreviations: CI, confidence interval; NPV, negative predictive value; PPV, positive predictive value. Bold font represents p values ≤0.05.

**Table 3.** Specificity and positive predictive values of MTB Plus, Ultima and Ultra for TB detection in comparison with MRS stratified by previous TB. Specificity of MTB Plus, Ultima and Ultra did not differ by previous TB status. Data are % (95% CI) and n/N

	No previous TB 288/498 (58)		Previous TB 210/498 (42)	
	Specificity	PPV	Specificity	PPV
**Smear microscopy**	98 (95, 99) 181/184	95 (87, 98) 60/63)	97 (93, 99) 134/138	92 (80, 97) 44/48
**MTB Plus**	94 (89, 97) 132/140	91 (83, 95) 79/87	96 (91, 99) 107/111	93 (84, 97) 57/61
			p=0.4363*	p=0.5628*
**Ultima**	83 (77, 87) 134/161	77 (68, 82) 88/115	87 (80, 91) 106/122	79 (68, 86) 60/76
			p=0.3962*	p=0.6944*
	**p=0.0028** ^#^	**p=0.0079** ^#^	**p=0.0096** ^#^	**p=0.0169** ^#^
**Ultra**	97 (93, 98) 178/184	94 (88, 97) 99/105	93 (87, 96) 129/139	86 (76, 92) 62/72
			p=0.1067*	p=0.0624*
	p=0.2820^†^	p=0.3557^†^	p=0.2199^†^	p=0.1698^†^
	**p<0.0001** ^†^	p=0.0002^†^	p=0.1111 ^†^	p=0.2523^†^

Within row p-values:

* Previous TB vs. No previous TB.

Within column p-values:

# MTB Plus vs. Ultima

† MTB Plus or Ultima vs. Ultra.

Abbreviations: CI, confidence interval; NPV, negative predictive value; PPV, positive predictive value. Bold font represents p values ≤0.05.
